# Plant Bioactive Compounds in Foods and Food Packages

**DOI:** 10.3390/foods13091419

**Published:** 2024-05-06

**Authors:** Ginés Benito Martínez-Hernández, Amaury Taboada-Rodríguez, Fulgencio Marin-Iniesta

**Affiliations:** 1 Food Safety and Refrigeration Engineering Group, Department of Agricultural Engineering, Universidad Politécnica de Cartagena, Paseo Alfonso XIII 48, 30203 Cartagena, Spain; ginesbenito.martinez@upct.es; 2 Agrosingularity, Calle Pintor Aurelio Pérez, 12, 30006 Murcia, Spain; ataboada@agrosingularity.com; 3 Group of Research Food Biotechnology-BTA, Department of Food Science, Nutrition and Bromatology, University of Murcia, Campus de Espinardo, 30100 Murcia, Spain

There has been growing interest in the use of numerous plant bioactive compounds (PBCs) in food and nutrition technology due to their properties that promote human health by reducing the risk of various serious diseases [1,2]. In addition to these effects, some of the PBCs that are present in food industry residues can be used as substitutes for toxic or xenobiotic chemical products, and thus can lead to significant positive environmental effects [3,4]. Specifically, some PBCs have technological effects and can replace chemically synthesized additives that are used in both food [4] and in compostable food packaging that does not include plastics of petrochemical origin [5].

Among the most important properties of PBCs we can list their antioxidant, antimicrobial, thickening, emulsifying, film-forming, prebiotic, and other effects that allow them to prolong the shelf life of foods and improve their safety and nutritional properties [3,6]. However, problems such as a strong taste, low solubility, high volatility, and instability make it difficult to apply them to food and food packaging. A large number of studies have attempted to avoid these problems with the use of new technologies for clean extraction, modification of the native properties of PBCs, and the application of PBCs to both food and food packaging [7,8,9].

We hope that this Special Issue will be useful to all readers as it provides new information on the applications of bioactive plant products in food and food packaging and the ways in which they can contribute to the production of healthier and safer foods. The following core areas of research are included in this Special Issue:

(1) Food additives can be obtained from synthetic or natural sources. Numerous scientific findings (including epidemiological studies like the contentious Southampton study published in 2007 and in vitro studies) have indicated that a number of synthetic additives may cause health problems for consumers. Hence, it is necessary to replace the chemical substances in food ingredients and food contact materials (packaging) with natural alternatives. Thus, the review [10] published in this Special Issue presents natural plant substitutes for common synthetic emulsifiers, colorants, flavourings, inhibitors of enzymes that degrade quality, antimicrobials, and antioxidants, together with an analysis of their likelihood of success. In addition, the requirement of a total lack of chemical additive migration from the packaging to the food is met by using plant bioactive compounds derived from plants (like plant essential oils) in active packaging.
(2) Plant essential oils (EOs) are PBCs with high antimicrobial and antioxidant activities and they have been well-known since ancient times. Nevertheless, the application of EOs as a substitute for synthetic chemical additives in both food and food packaging is highly limited due to their characteristic organoleptic properties (mainly aroma and flavour). The proper combination of different EOs may mask these organoleptic aspects, and may also even have a synergistic effect on their antimicrobial and antioxidant properties. In that sense, Cava Roda et al. [11] studied the in vitro antimicrobial effects of different EOs (cinnamon bark, cinnamon leaves, and clove) and their main components (cinnamaldehyde, eugenol, and vanillin), either individually or in combination, against *Listeria monocytogenes* and *Escherichia coli* O157:H7. The combinations of vanillin/clove EO and vanillin/cinnamon bark EO showed the most synergistic antimicrobial effect (assessed with a two-dimensional checkerboard assay and the isobologram methods), while their main components showed lower antimicrobial activities [11].
(3) The feasibility of substituting a common synthetic antioxidant (butylhydroxytoluene) with a natural PBC was studied by Jonušaite et al. [12]. As previously described, the combination of EOs may lead to enhanced antioxidant and antimicrobial activities against a wide spectrum of microorganisms. In that sense, Jonušaite et al. [12] compared the antioxidant activity of butylhydroxytoluene and EOs (black elderberry flower extract, oregano EO, and clove EO) on the prevention of lipid peroxidation in fish products (salmon burger) during cold storage. The oregano EO/flower extract showed the same antioxidant activity as the chemical antioxidant (butylhydroxytoluene), effectively controlling the microbial growth of the fish product during cold storage [12].
(4) As previously mentioned, the use of EOs in food and food packaging is very limited due to the flavours/aroma related to the high doses needed (to achieve the desired antimicrobial/antioxidant properties). However, EOs in the vapour phase (with lower doses) are more effective than EO-based edible coatings in the liquid phase (which need higher doses). The effectiveness of a vapour EO mix (eugenol and bergamot and grapefruit EOs) injected into the atmosphere of plastic trays including slice mushrooms on the product’s quality during cold storage was studied [13]. The injection of the vapour EOs highly controlled the reduction in the quality of the sliced mushrooms owing to their enzymatic inhibition and high bacteriostatic effects.
(5) Active packaging with antimicrobial and/or antioxidant PBCs allows the shelf life of a packaged product to be extended. In that sense, the effect of the incorporation of trans-2-hexenal (a PBC found in strawberries with excellent antifungal properties) in pads allocated in the bottom of plastic trays with fresh strawberries was studied on the fruit quality during cold storage [14]. The mould growth in the fresh strawberries was completely inhibited with the active packaging, although the firmness and colour of samples were affected by the trans-2-hexenal released from the active package [14].
(6) Propolis extract has significant antimicrobial and antioxidant effects, among a long list of its other health-promoting properties that are widely known. In addition, chitosan, a bioactive compound found in high quantities in shellfish shells, has several interesting properties, although it is mostly known for its high antimicrobial activity. In that sense, Vargas-Romero et al. [15] studied the antimicrobial activity of active packaging (films made of low-density polyethylene) including Colombian propolis extract and chitosan, which are both bioactive compounds with high antimicrobial and antioxidant properties, on the quality of fresh pork loins. The meat packaged with these active films showed better colour and lipid stability, as well as better bacterial growth, during the studied cold storage [15].
(7) The antioxidant and antimicrobial properties of bioactive compounds may even be enhanced when functionalized with other bioactive compounds. Hence, the antimicrobial and antioxidant activity of chitosan was enhanced when it was functionalized (using the carbodiimide-mediated grafting method) with hydroxycinnamic acids (p-coumaric acid, caffeic acid, and ferulic acid), and the final functionalized bioactive compound was used during the fabrication (solvent casting method) of an innovative active film [16]. The functionalization of chitosan improved the film’s properties (mechanical strength, thermal stability, and UV light barrier and water vapour barrier abilities) and led to higher antioxidant and antimicrobial activities, which may extend the shelf life of meat products [16].
(8) A new active film based on an ethylene vinyl alcohol copolymer supplemented with cinnamaldehyde (the major bioactive compound of the cinnamon EO) was developed using a hybrid technique [17]. The cinnamaldehyde addition led to a UV-blocking effect in the film and a plasticizing effect. In addition, the active film showed radical scavenging and antimicrobial activity (in vitro assay against *Penicillium expansum*) due to the cinnamaldehyde incorporation [17].
(9) The effectiveness of active packaging including EOs may be increased through the encapsulation of EOs and their incorporation into the active packaging, resulting in a controlled release of small quantities of EOs and avoiding the characteristic flavours/aromas of EOs. In that sense, the encapsulation of EOs with cyclodextrins through the formation of inclusion complexes was approached by López-Gómez et al. [18]. In particular, an EOs mix (carvacrol/oregano EO/cinnamon EO) was nanoencapsulated in *β*-cyclodextrin and included in active paper sheets for the conservation of flat peaches during cold storage [18]. These authors found that active paper sheets controlled the production of ethylene in the flat peaches, maintained fruit quality, and even increased the PBC contents of this fruit.
(10) There is significant interest in the development of different types of edible coatings and films that can provide a protective barrier against agents such as pathogenic or spoilage organisms, moisture, oxygen, and carbon dioxide, and that can also improve the sensory and mechanical properties of stored fruits. The effect of edible starch films with binary combinations of PBCs (thymol and carvacrol) in controlling the plant pathogen *Colletotrichum gloeosporioides* in mango and papaya fruits has been studied. Two binary mixtures, previously selected through in vitro studies, were used to coat the fruits, and a longer shelf life at 20 °C was obtained, concerning the fruits’ colour, firmness, and maturity index. In addition, inhibition of the growth of *C. gloeosporioides* with a significant reduction in lesions on the assayed fruits was observed.
(11) Food processing may also lead to a side effect consisting of the loss of PBCs from the treated food products. For example, the vacuum-cooling technology used as an efficient pre-refrigeration technique for leafy vegetables may also lead to the loss of PBCs, as has been found to occur for the volatiles found in aromatic herbs. Nevertheless, innovative technology was proposed consisting of the application of EO vapours at the end of the vacuum cooling of fresh culinary herbs, allowing the aromatic recovery of these plant volatiles [19]. Hence, the aroma recovery of culinary herbs was successfully validated at an industrial level with this innovative technology, reaching total volatile recoveries of 2-5-fold, for example, in parsley and dill [19].
(12) The extractability of PBCs is an important aspect of supplying these bioactive compounds of technological interest for their application in food and food packaging. Sustainable/green technologies are being explored for this purpose, and also to avoid possible PBC degradation using so-called non-thermal technologies. The use of high hydrostatic pressure was first proposed in the eighteenth century as a process to preserve food, although its capacity to extract bioactive compounds with high-efficiency rates is also well known. Interestingly, recent innovation points to the use of high hydrostatic pressure with multi-pulse cycles. In that sense, the extractability of phenolic compounds and carotenoids from carrots increased with multi-pulse high hydrostatic pressure treatment, and their phenolic accumulation also increased after a short storage period of 48 h [20].
(13) Artichokes are vegetables that are appreciated for their high contents of several bioactive compounds such as phenolic compounds (e.g., hydroxycinnamic acid) and carotenoids (e.g., luteolin derivatives), although little is known about the effect of the order of the harvest of the flower heads of the plant (harvests of the same artichoke plant are conducted at different times leading to main, secondary or tertiary heads) on these bioactive contents. The effect of treatment with the phytohormone gibberellic acid on these bioactive compounds was also studied [21]. The highest bioactive compound contents were observed in the tertiary heads, although the gibberellic acid treatment showed different effects: increased luteolin derivatives levels but decreased hydroxycinnamic acid content.
(14) The health-promoting effects of PBC must also be ensured, along with high digestibility and bioaccessibility after consumption. In that sense, the digestibility of fatty acids and the bioaccessibility of phenolic compound extracts (as oil-in-water emulsions) of avocado by-products (peel and seeds) were studied [22]. The bioaccessibility of phenolic compounds from the avocado peel (which were higher than their contents in the avocado seed) was higher than those from the avocado seeds. In addition, the low levels of methoxypectin and the avocado extracts both increased the digestibility of the fatty acids.
(15) Several studies have been carried out on the applications of EOs in aquaculture due to their various anaesthetic, antioxidant, and antimicrobial properties, among others [23]. The application and effects of nanoencapsulated clove EO in *β*-cyclodextrins, incorporated into the stunning water, on the response to stress and organoleptic properties of fish (Atlantic salmon, European sea bass, Nile tilapia and rainbow trout) were studied by López-Cánovas et al. [23]. Encapsulated clove EO led to a significant reduction in the time needed to induce anaesthesia in fresh and marine water fish at different temperatures, with a decrease in the fish’s stress and a prolongation of their shelf life [23].

## Data Availability

Not applicable.
